# Evaluating a digital health ecosystem for asthma and air quality: an early feasibility study based on clinicians and patient-reported measures

**DOI:** 10.3389/fdgth.2026.1766023

**Published:** 2026-02-25

**Authors:** Sergio-Vidal Escalona-López, Zouhair Haddi, Luis Lores Obradors, Joan Vinyets Rejón, Anne Sophie Gresle, Sabina De Rosis, Rosana Hernando Salvador

**Affiliations:** 1 Pneumology Department, Hospital General Parc Sanitari Sant Joan de Déu, Barcelona, Spain; 2 Digital Health and Gas Sensing Department, NVISION Systems and Technologies, Barcelona, Spain; 3 Facultat de Medicina i Ciències de la Salut, Universitat de Barcelona, Barcelona, Spain; 4 Àrea Experiència de Pacient, Hospital Sant Joan de Déu, Barcelona, Spain; 5 Patient Experience Observatory, Hospital Clínic de Barcelona, Barcelona, Spain; 6 Science with and for Society Hub, ISGlobal, Barcelona, Spain; 7 Department of Law, Economics and Human Science, University Mediterranea of Reggio Calabria, Reggio Calabria RC, Italy; 8 Institute of Management & Health Science Interdiscipliary Center, Sant'Anna School of Advanced Studies, Pisa PI, Italy

**Keywords:** air quality, asthma, digital health, early feasibility study, eHealth monitoring Ecosystem, patient-reported experience measures (PREMs), remote monitoring

## Abstract

**Introduction:**

Asthma remains a major global health burden, particularly in contexts of poor air quality where managing moderate and severe diagnosis ideally requires continuous, coordinated care and remote monitoring beyond episodic clinic visits. In this setting, this early feasibility study evaluates a novel digital health platform, developed as part of an integrative eHealth Monitoring Ecosystem for patients diagnosed with moderate or severe asthma, aimed at supporting personalised, continuous asthma care through remote physiological and air quality monitoring.

**Methods:**

The system was conceptualized, designed, refined, and assessed throughout the study, integrating wearable physiological sensors, individualised portable air quality monitors, and a digital interface with separate portals for patients and clinicians. Thirteen patients and seven clinicians participated in a six-month pilot, interacting with the platform through their respective user interfaces. Their views on system use, care processes, emotional responses, overall satisfaction, and the anticipated integration of the system into routine care were assessed using a 26-item Likert-scale Patient-Reported Experience Measures (PREMs) questionnaire, administered to both patients and clinicians and organized into five domains: user experience (including both patients and clinicians), patient experience, emotional experience, overall satisfaction, and future implementation expectations. An additional open-ended comment (item 27) was included and analyzed using inductive qualitative methods in NVivo, with themes aligned to the same five clusters.

**Results:**

The findings indicate a high level of positive experience with the eHealth Monitoring Ecosystem across all PREMs domains, with patients requesting more clinical and technical information and clinicians identifying the need for enhanced functionality and additional tools.

**Conclusion:**

These results support the feasibility and acceptability of the eHealth Monitoring Ecosystem for moderate and severe asthma management and suggest directions for further refinement, broader clinical evaluation, and potential adaptation to other chronic disease monitoring contexts.

## 1 Introduction

Asthma remains a major global health burden, particularly in areas with high levels of air pollution, where managing moderate and severe asthma requires continuous, coordinated care. In response to this clinical burden and to the practical challenges of integrating systematic environmental monitoring into routine care, our team developed an eHealth Monitoring Ecosystem (eHME) that combines physiological and environmental monitoring with a digital interface for patients and clinicians. Among pollutants, ambient exposure to particulate matter (${\mathrm{PM}}_{2.5}$) and nitrogen dioxide (${\mathrm{NO}}_{2}$) shows a particularly strong association with both the incidence and severity of asthma worldwide [1, 2]. Asthma is estimated to affect over 339 million people worldwide, and its global prevalence continues to rise, with tens of millions of new cases projected in the coming years [3, 4]. In particular, moderate and severe asthma poses persistent challenges for both patients and healthcare systems due to the need for ongoing, multidisciplinary management [5, 6]. Recent advances in digital health, including web-based portals, mobile technologies, and asynchronous communication tools, offer an opportunity to transform asthma care through sustained, personalised monitoring beyond clinic walls [7]. However, the integration of digital health technologies into routine clinical practice continues to face significant barriers, including usability challenges, limited patient engagement, lack of interoperability, and difficulties in synthesizing clinical, environmental, and experiential data within existing healthcare infrastructures [8, 9].

To better understand how patients and clinicians perceive these technology-supported models of care, patient-reported experience measures (PREMs), traditionally used to assess patients’ experiences with healthcare delivery, are increasingly being adapted to evaluate user experience in digital health interventions. This broader use of PREMs enables the systematic capture of structured insights into usability, satisfaction, and service quality within technology-supported care [10, 11]. Unlike clinical or physiological outcomes, PREMs capture patients’ subjective experiences of healthcare delivery, including elements such as communication, accessibility, coordination, and perceived value of digital tools. According to international health quality frameworks [12, 13], the use of PREMs allows healthcare systems to evaluate not only what care is delivered but also how it is experienced by the user. In the field of digital health, their application is growing rapidly. A recent systematic review highlighted the increasing adoption of PREMs in the evaluation of telemedicine and mobile health applications, particularly for assessing user engagement, satisfaction, and usability in real-world contexts [11]. Nevertheless, challenges remain in standardising PREMs instruments and fully integrating patient-reported experiences into both clinical evaluation and technology development processes. Recent evidence further shows that PREMs can enhance patient-centred evaluation of digital interventions, support continuous quality improvement, and contribute to more responsive system design [14], while challenges such as integration into existing workflows and variability in implementation strategies highlight the need for careful application of PREMs in real-world settings [15]. These considerations support the relevance of using PREMs in studies like the present one, where the user perspective is a core component of feasibility assessment. However, many studies on digital health still focus mainly on technical performance or clinical results, often overlooking how patients actually experience these tools and how clinicians incorporate them into day-to-day practice. Recent reviews have highlighted that usability issues, lack of integration into clinical workflows, and insufficient attention to user engagement remain common limitations in digital health studies, particularly in the context of chronic disease management such as asthma [9, 16].

To address these gaps, we developed and evaluated a novel eHealth Monitoring Ecosystem (eHME) for the management of moderate and severe asthma. The eHME integrates wearable physiological monitoring, individualised air-quality exposure assessment, and a multilingual digital platform with tailored interfaces for both patients and clinicians, supported by a structured clinical follow-up protocol in hospital settings. By centring user experience (which includes both patient and clinician perspectives) through a comprehensive PREMs questionnaire and inductive qualitative analysis, this Early Feasibility Study (EFS) aims to assess the practicality, acceptability, satisfaction, and readiness for broader clinical adoption of the eHME and future scale-up, as evaluated from the perspectives of both patients and clinicians.

## 2 Methods

The study adopted an Early Feasibility Study (EFS) design to support the initial clinical evaluation of the proposed eHME for moderate and severe asthma in a real-world hospital setting [17]. The primary objective was to assess practical deployment and integration within standard asthma care pathways, as well as overall user experience from both patient and clinician perspectives. This was an observational, cross-sectional, mixed-methods EFS, in which a structured PREMs questionnaire served as the main instrument for evaluating participant experience.

Within this framework, patient and clinician experience with the eHME was assessed using PREMs to capture structured feedback on usability, satisfaction, engagement, and potential barriers to adoption [14, 15]. While PREMs do not directly measure acceptance, the experiential dimensions they capture (usability, perceived value, or emotional response) can inform perceived suitability for future clinical use. Accordingly, PREMs were used to evaluate how patients and clinicians experienced the eHME in routine care, allowing the ecosystem to be assessed not only as a technological prototype but also as a user-centred component of everyday asthma management.

### 2.1 eHME development and implementation

The eHME was developed and gradually refined by clinicians, engineers, and pilot healthy volunteers to ensure acceptable technical performance and usability in real-world conditions. The eHME design was informed by prior work on individualised air quality exposure assessment in asthma, including platforms integrating wearable environmental sensing with digital tools such as AirPredict [18].

**Figure 1:**
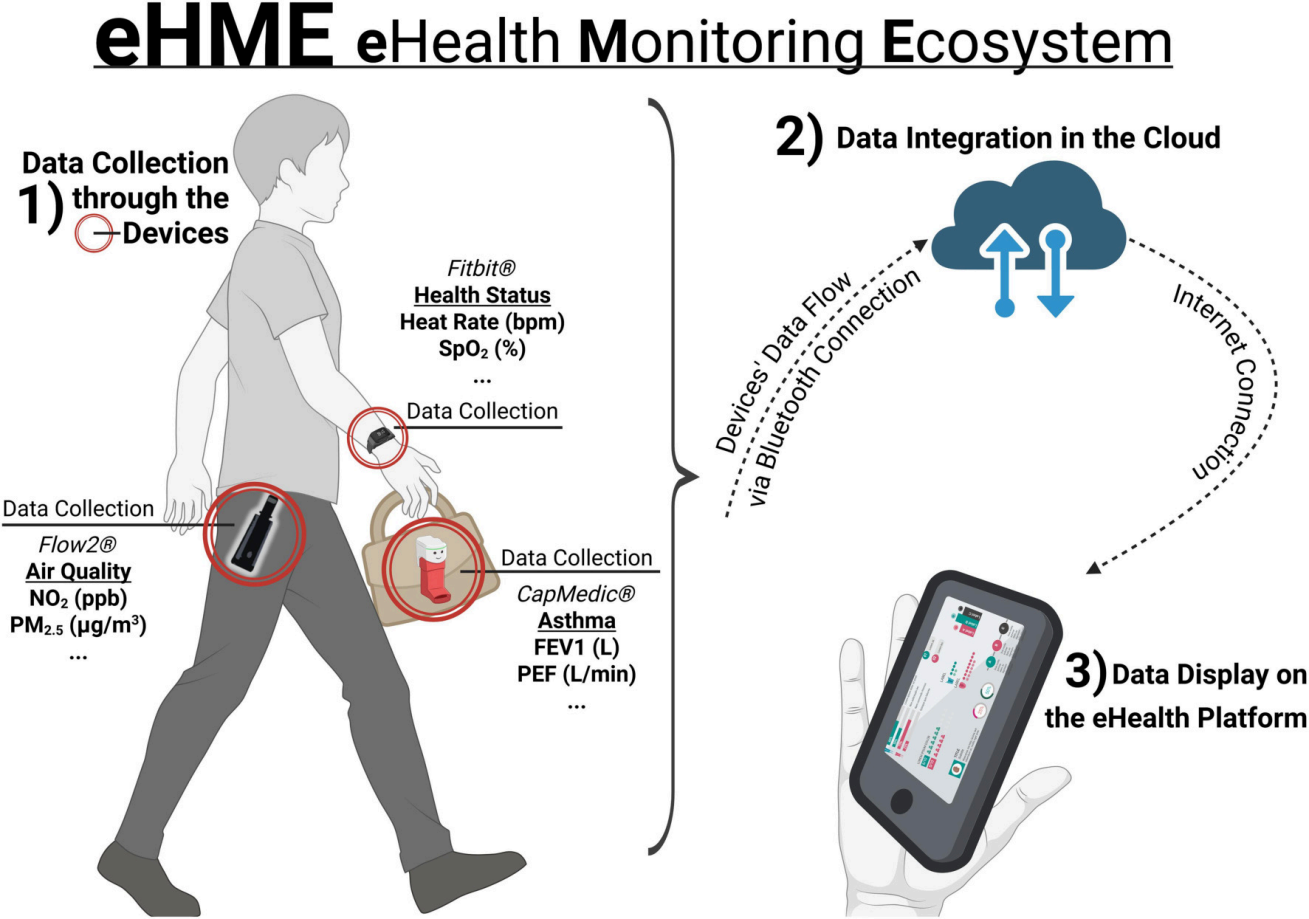
Schematic representation of the data flow within the eHealth Monitoring Ecosystem (eHME). The system integrates three data sources: Fitbit® (physiological status), CapMedic® (asthma-related lung function metrics), and Flow2® (environmental air quality parameters). Data are transferred via Bluetooth to the participant’s smartphone, uploaded to the cloud for integration and processing, and displayed on the eHealth platform. *Created with BioRender.com*.

Data collection relied on wearable and portable sensors, including an individual air quality monitor (Figure 1). The air quality device was selected to address the spatial and temporal limitations of stationary monitoring by capturing high-resolution personal exposure linked to daily activity patterns [19]. Such personal monitoring can reveal substantial within-person exposure variability across microenvironments and short-term peaks that may be missed by fixed stations [20].

The eHME integrated three data streams via distinct devices: Fitbit for physiological data (including heart rate and ${\mathrm{SpO}}_{2}$), CapMedic for asthma-specific parameters (such as ${\mathrm{FEV}}_{1}$ and peak expiratory flow), and Flow2 for individualised environmental exposure metrics (notably ${\mathrm{NO}}_{2}$ and ${\mathrm{PM}}_{2.5}$). The ecosystem included a multilingual platform available in Spanish and Catalan to reflect the linguistic context of the hospital’s local community.

To improve accessibility and user-friendliness, the front end was designed to display health and environmental information with minimal cognitive load and limited disruption to daily routines [21]. In line with established mHealth usability heuristics (clear navigation and minimalist layouts) [22], the interface avoided overloaded screens and excessive notifications; unobtrusive, user-centred design has been associated with better retention [21].

All data collected through the eHME were pseudonymised at source and uploaded from the smartphone to a secure cloud-based infrastructure with controlled access. No directly identifiable personal data were stored within the platform, and study data were handled using participant codes in accordance with data protection regulations. Access to the clinician-facing web application was restricted to authorized health professionals involved in the study through individual credentials. The platform was designed to support longitudinal data visualization and retrospective review within the context of this early feasibility study, and it was not intended to function as a real-time clinical monitoring system. Accordingly, no predefined algorithmic thresholds or automated alerts were implemented at this stage of development, and notifications were limited to operational reminders such as self-measurements or daily questionnaire completion. This design choice aligns with the exploratory aims of the EFS and the non-medical device status of the platform, to avoid inappropriate or potentially misleading clinically actionable notifications. Throughout the study period, clinical decision-making and urgent care management remained under routine clinical practice and standard care pathways.

**Figure 2:**
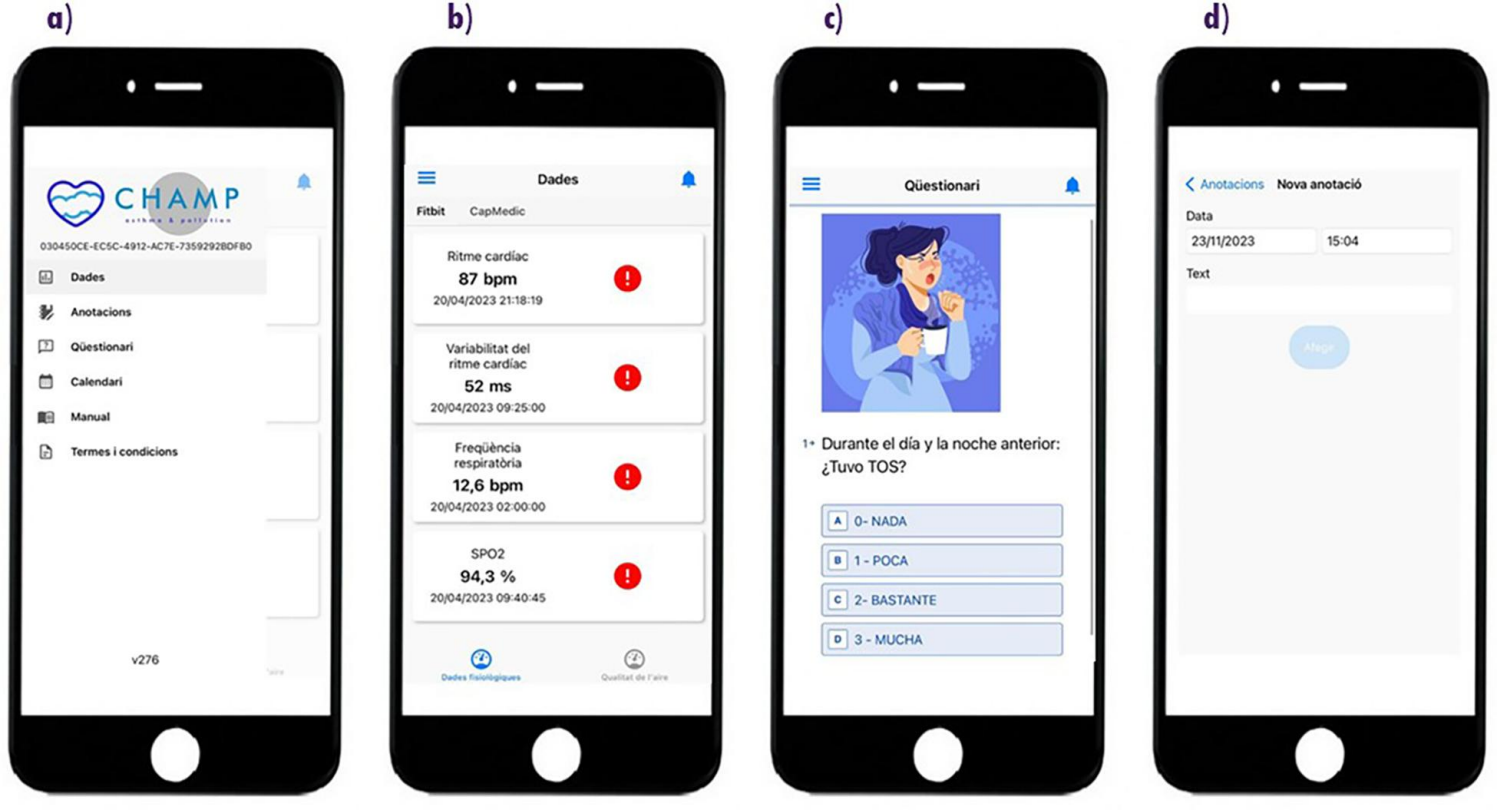
Screenshots of the eHME smartphone application. **(a)** Home screen with main navigation (data, questionnaires, annotations, calendar, user manual, and terms). **(b)** Physiological data display from Fitbit® (heart rate, respiratory rate, and oxygen saturation). **(c)** Symptom questionnaire interface (like cough intensity). **(d)** Annotation module for manual, timestamped notes.

Consistent with these design principles, the patient-facing application was designed to support routine interaction with the eHME system, and from the user’s perspective its main components were:

**Patient-facing smartphone application:** As shown in Figure 2, patients accessed the eHME smartphone app daily to interact with several key features. The home screen provided an overview of their current physiological parameters (such as heart rate, respiratory rate, and oxygen saturation) obtained from the Fitbit device and also displayed air quality indicators, including NO2 and PM2.5 levels from the Flow2 device. Patients could also access symptom questionnaires, including daily patient-reported outcome measures (PROMs), and navigate to their visit calendar, user manuals, or privacy policy documents. The interface also allowed patients to add manual annotations, for example describing specific symptoms, exposures, or situations that might be relevant for later clinical interpretation. To further enhance usability and engagement, the eHME enabled bidirectional annotations, allowing both patients and clinicians to contextualize data entries, such as symptom spikes or environmental exposures.

**Figure 3:**
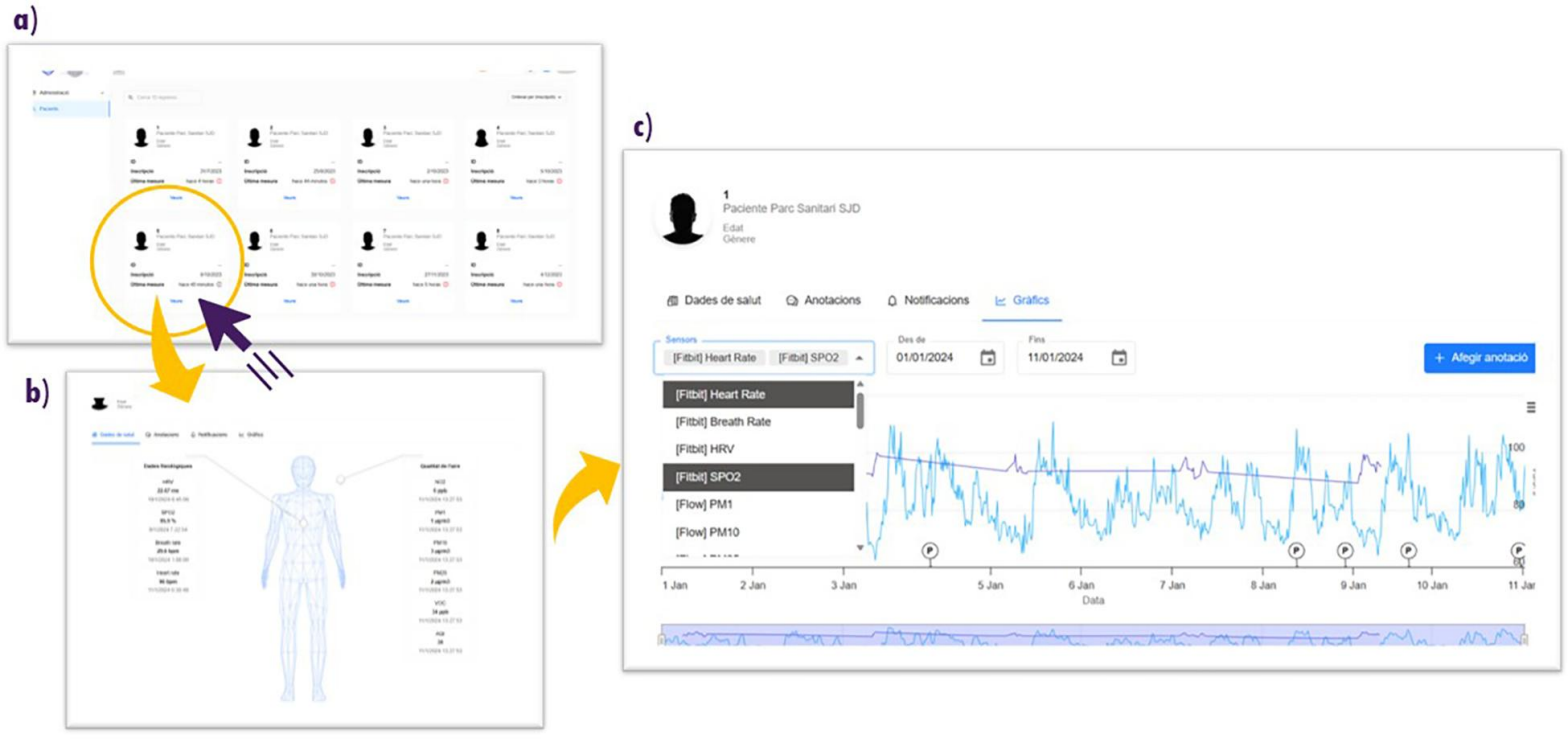
Clinician web interface of the eHME platform. **(a)** Patient overview list with basic information. **(b)** Individual patient profile summarising physiological and environmental data. **(c)** Longitudinal visualisation of sensor data (like heart rate, ${\mathrm{SpO}}_{2}$), with parameter selection, time-range customisation, and event annotation.

**Clinician web application:** As shown in Figure 3, clinicians accessed a dedicated web application to monitor patient data over time during the study period. The main view presented a list of enrolled patients with basic clinical information and provided direct access to individual dashboards summarising each patient’s physiological and environmental data. Clinicians could then review daily sensor readings, visualize longitudinal trends in parameters such as heart rate, oxygen saturation, and pollution exposure, and annotate events using the same annotation feature available in the patient app when relevant. Overall, this interface allowed them to remotely monitor patients’ status, contextualize symptoms, and support ongoing clinical assessment outside routine visits.

The system was designed to enable continuous collection and contextualization of physiological and environmental data, while also supporting patient engagement and clinician oversight. It aims for a robust foundation for personalised asthma management and presents a scalable model for future applications in moderate and severe asthma care and the monitoring of other chronic or mental health conditions.

### 2.2 Clinical study design and participants

The clinical study was conducted as a real-world Early Feasibility Study (EFS) and structured as a six-month, mixed-methods observational pilot to evaluate the practicality, safety, and perceived experience of a novel eHME for the management of moderate and severe asthma. Thirteen adult patients aged 22 to 85 years with a confirmed diagnosis of moderate or severe asthma were recruited from the Pneumology Department at Hospital General Parc Sanitari Sant Joan de Déu (Sant Boi de Llobregat, Barcelona, Spain). Labile asthma was not used as an exclusion criterion. To ensure clinical stability at enrolment, participants had not experienced an acute exacerbation in the month prior to inclusion. Potential candidates were identified during routine outpatient consultations or through a proactive recruitment campaign developed with the hospital’s Communications Department, including dissemination via social media, the hospital website, and printed materials (posters and leaflets). All interested patients received detailed study information and provided written informed consent prior to enrolment.

Along with the thirteen patients, seven clinicians participated in the evaluation of the digital platform. All participating clinicians were medical doctors from the same Pneumology Department, routinely involved in the clinical management of patients with moderate or severe asthma.

The clinical protocol was designed to maximize data comprehensiveness while minimizing participant burden. Each patient completed a total of seven scheduled evaluations over the six-month study period: four in-person hospital visits (at baseline, month 2, month 4, and month 6) and three telephone follow-ups scheduled between these timepoints. The study did not modify or replace routine clinical care. If participants experienced asthma worsening or required urgent care during the monitoring period, they were instructed to follow their usual clinical pathways (contact their usual care team or attend emergency services as appropriate). Any such events were documented during the subsequent scheduled study follow-up visit to contextualise the monitoring data. In the event of hospital admission, clinical management would have followed standard hospital care pathways independently of the study, and eHME data collected during that period would have been paused or interpreted in that clinical context.

During in-person visits, patients underwent standard clinical assessments, including spirometry, physiological measurements, and blood sampling. A set of validated PROMs was also administered to evaluate symptoms, wellbeing, and psychological status. These assessments were used to complement the digital and experiential data collected through the eHME.

On day 0 of the study, each patient was provided with the three study devices (see Figure 1), written instructions, and formally signed the informed consent documentation. During this visit, participants received in-hospital training from the research team on device handling and on how to perform the required self-measurements depending on the device. Although patients were already familiar with the inhaler device (CapMedic), a reinforcement of its correct use was provided by the clinical team to ensure optimal performance during the first study visit. In addition, specific instructions were provided for the Fitbit and Flow2 devices included in the study to support correct use and ensure data quality. Throughout the study period, patients used the wearable physiological sensors, a portable air quality monitor, and the patient-facing eHealth application on a daily basis (see Figure 1 for the device setup and Figures 2, 3 for the patient and clinician interfaces, respectively), enabling continuous, individualised collection of physiological and environmental data.

### 2.3 Questionnaire administration

To evaluate the responsible introduction of the eHME among both patients and clinicians, a structured PREMs questionnaire was administered to all participants at the end of their voluntary involvement in the EFS. The instrument consisted of 27 items (26 Likert-scale items rated on a 1-to-7 scale and one additional free-text item), as detailed in Table 1. For this study, the full 27-item questionnaire was administered in Spanish and Catalan, ensuring linguistic and cultural appropriateness for the local population. These translations were reviewed internally for clarity and consistency, although no formal back-translation process was applied.

**Table 1:** PREMs questionnaire items grouped by thematic cluster (UX, PX, EX, FIX, and SAT). Items are shown as English translations of the Spanish and Catalan versions administered in this study; Q27 is an open-ended free-text item.

Cluster	Item	Question
UX	Q6	The eHME app is easy to use.
UX	Q7	The eHME app has an interface that is easy to understand (user-friendly).
UX	Q8	The eHME app requires little effort to access information.
UX	Q9	In the eHME app, the steps to reach information are logical; it’s hard to get lost.
UX	Q10	In the eHME app, you encountered problems when trying to access it. (Reverse-coded)
UX	Q11	The eHME app requires mental effort to use. (Reverse-coded)
UX	Q12	The eHME app has many errors. (Reverse-coded)
UX	Q13	In the eHME app, when you get lost, it’s difficult to retrace your steps. (Reverse-coded)
UX	Q14	The eHME app is easy to learn how to use.
UX	Q15	The app is quick to learn how to use.
UX	Q16	In the eHME app, it took little effort to learn navigation.
UX	Q17	The eHME app is easy to use, even with no prior knowledge.
UX	Q18	For the eHME app, written instructions are necessary. (Could indicate need for improvement)
UX	Q26	You like the appearance of the eHME app.
PX	Q1	The eHME app helps you better understand your asthma.
PX	Q2	The eHME app helps you better manage your asthma.
PX	Q3	The eHME app gives peace of mind knowing your doctor is aware of your status.
PX	Q4	The eHME app makes you feel secure by providing asthma and pollution info.
PX	Q5	The eHME app makes you happy to have info readily available.
PX	Q21	The eHME app allows you to carry on with normal life (flexibility).
EX	Q24	You feel comfortable using the eHME app.
EX	Q25	You feel safe using the eHME app.
FIX	Q22	You would like the eHME app implemented in clinical settings.
FIX	Q23	You’d be delighted to use the eHME app if it helps your doctor personalize treatment.
SAT	Q19	Overall, you are satisfied with the eHME app.
SAT	Q20	You would recommend the eHME app to friends/family.
–	Q27	Do you have any comments, feedback, criticism, advice, or suggestions regarding the eHME app?

The PREMs questionnaire used in this study was primarily based on the validated MAUQ for interactive patient-facing applications developed by Zhou et al. [23], which includes 21 items covering ease of use, information arrangement, and perceived usefulness. To tailor the instrument to the specific clinical context of this study, the research team added five items (Q1–Q5) addressing patient understanding of asthma, sense of safety, and confidence in the clinician–patient relationship, with a particular focus on disease self-management and perceived clinical integration. The remaining Likert-scale questions (Q6–Q26) correspond to the original MAUQ items, and a final free-text item (Q27) was included to allow participants to comment freely on their experience. All questionnaire items used in this study are listed in Table 1.

In this study, we distinguish between *user experience* and *patient experience*. The term *user* refers to any individual interacting with the eHME, including both patients and clinicians. In contrast, the *patient experience* cluster focuses specifically on experiential elements related to clinical care and personal health management. This distinction allows us to analyze general usability and interface design separately from how patients perceive the platform’s contribution to their understanding, autonomy, and wellbeing. This approach is consistent with broader work on patient-centred performance measurement, where experiential measures such as PREMs are conceptually distinguished from other quality indicators [24].

The items were organized into five thematic clusters, each designed to assess a distinct experiential domain: User Experience, Patient Experience, Emotional Experience, Future Integration Expectations, and Satisfaction (for further information, see Table 1).

The five experiential clusters were defined by the research team specifically for this study, drawing on existing conceptual work on digital health evaluation and person-centred performance measurement, particularly literature on the integration of PROMs and PREMs and on co-production in healthcare systems [25]. Rather than adopting a single validated framework, we selected and combined dimensions that are frequently described in the digital health and patient experience literature to construct a multidimensional structure suitable for evaluating both patient and clinician interactions with the eHME. This approach was intended to ensure that the questionnaire captured domains that are relevant for practice, interpretable by clinicians, and aligned with real-world implementation contexts.

Below, we describe each cluster in detail to clarify the experiential dimensions they assess:

**User experience (UX):** This cluster comprises 14 items focused on usability, accessibility, learnability, and visual design. The questions evaluate how easily users can operate the eHealth platform, access and interpret information, and navigate its structure. Items related to error handling, required effort, and the need for external instructions provide insight into cognitive load and system intuitiveness, while the visual appearance item captures aesthetic aspects of interaction. These usability dimensions are commonly linked to satisfaction, efficiency, and engagement in digital health technologies [26].

**Patient experience (PX):** The 6 items in this cluster assess how the eHME supports patients’ understanding of their condition, self-management capabilities, and active participation in care. The questions address both the practical role of the app in day-to-day asthma management and experiential aspects such as perceived support and flexibility in daily life. mHealth tools that provide clear information and personalised guidance have been associated with improved engagement in self-management contexts [27].

**Emotional experience (EX):** This cluster includes two items assessing users’ emotional responses to the app, specifically feelings of comfort and safety. These affective dimensions are relevant in healthcare settings where trust and perceived reliability can shape engagement with digital tools [28].

**Future integration expectations (FIX):** This cluster consists of two future-oriented items exploring participants’ views on potential clinical integration of the eHME, including willingness to use it in routine care and perceived value for personalised treatment. Anticipated adoption of digital health tools is often shaped by expectations of clinical usefulness and alignment with individual care goals [29].

**Satisfaction (SAT):** This cluster includes two items assessing participants’ overall evaluation of the eHME and their willingness to recommend it to others. Satisfaction is commonly used as a summary indicator in digital health evaluation and can influence continued use and recommendation of a platform [30].

It is important to distinguish between *experience* and *satisfaction*, as they capture related but distinct constructs. While experience refers to what actually happened during the interaction with the system (e.g., usability, communication, emotional response), satisfaction reflects an overall evaluative judgment, influenced not only by the experience itself but also by prior expectations and personal preferences [31]. Including both domains allows for a more comprehensive understanding of how users perceived the eHME and their willingness to recommend or continue using it.

Together, these five clusters supported a multidimensional assessment of participants’ experience with the eHME. The questionnaire was implemented in Typeform and accessed through the eHME platform at study completion. After the 26 Likert-scale items, participants were invited to answer item Q27, an open-ended free-text question to provide additional qualitative feedback.

## 3 Results

### 3.1 Quantitative PREMs analysis

**Figure 4:**
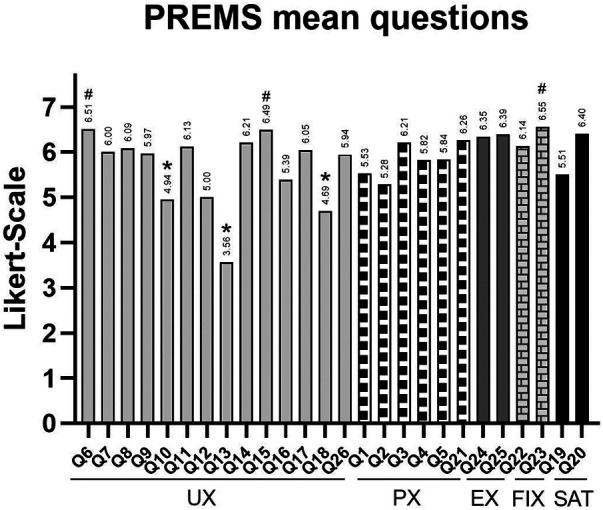
Mean scores for individual PREMs items (1–7 Likert scale), grouped by thematic cluster. *indicates lower-scoring items and # highlights the highest-rated item within each cluster.

Aggregated analysis of the 26-item PREMs questionnaire, grouped into the five clusters described in Section 2.3, showed consistently favourable ratings across clusters and participant groups (patients and clinicians). As shown in Figure 4, 25 of 26 items scored above the midpoint (4/7), indicating favourable responses. The only exception was item Q13 from the UX cluster, which received a mean score of 3.56. Other lower-scoring items within the UX cluster included Q18 (4.69) and Q10 (4.94), highlighting potential areas for usability improvement. The highest-rated items were Q23 (6.55) from FIX, followed by Q6 (6.51) and Q15 (6.49) from UX, reflecting strong support for future clinical integration and ease of use.

**Figure 5:**
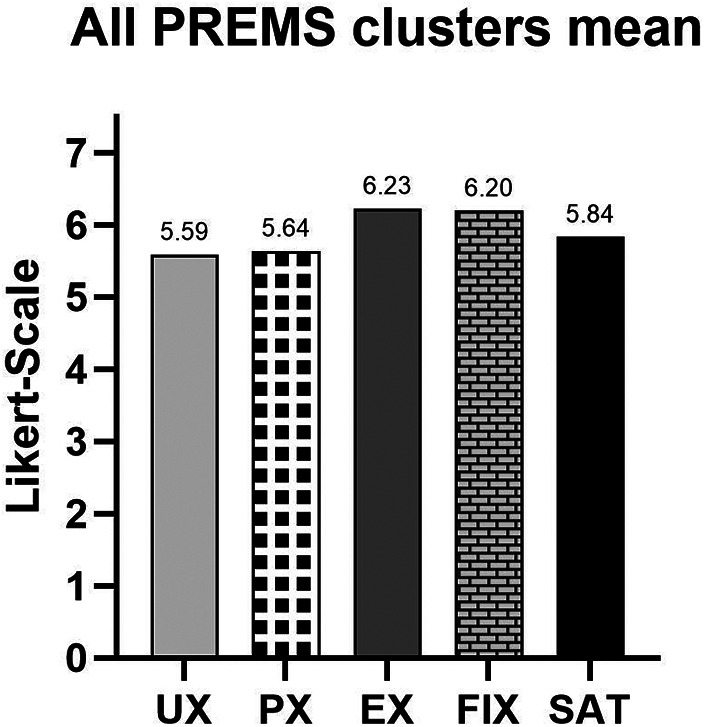
Mean PREMs scores by thematic cluster (UX, PX, EX, FIX and SAT) on the 1–7 Likert scale; EX and FIX showed the highest average ratings.

Across clusters (Figure 5), EX and FIX obtained the highest average scores (6.23 and 6.20), whereas UX had the lowest average score (5.59), still within a favourable range.

- **User experience (UX):** The UX cluster achieved a mean score of 5.59 out of 7. The highest-rated items within this cluster were Q6 (6.51) and Q15 (6.49), reflecting positive perceptions of ease of use and interface clarity. The lowest score was for Q13 (3.56), the only item in the entire questionnaire to fall below the neutral midpoint, indicating some difficulty in retracing navigation steps. Other notably lower-scoring items included Q18 (4.69) and Q10 (4.94), both pointing to areas for improvement in usability and onboarding clarity.
- **Patient experience (PX):** This cluster, composed of five items, yielded a mean score of 5.64. Q21 was the highest-rated item (6.26), indicating strong appreciation for the flexibility offered by the platform, while Q2 received the lowest score (5.28), suggesting room for enhancement in specific aspects of asthma self-management support.
- **Emotional experience (EX):** Scoring the highest among all clusters, the EX domain achieved a mean score of 6.23. Individual item scores were Q24 (6.35) and Q25 (6.39), showing consistently strong emotional reassurance and trust in the platform.
- **Future implementation expectations (FIX):** This cluster received a mean score of 6.20. The two items in this cluster, Q22 and Q23, scored 6.14 and 6.55 respectively, indicating strong willingness to adopt the eHME in routine practice, especially if it contributes to personalised treatment.
- **Satisfaction (SAT):** The satisfaction cluster achieved a mean score of 5.84, indicating overall approval of the platform. Q20 scored particularly high at 6.40, while Q19 received a slightly lower score of 5.51.

**Figure 6:**
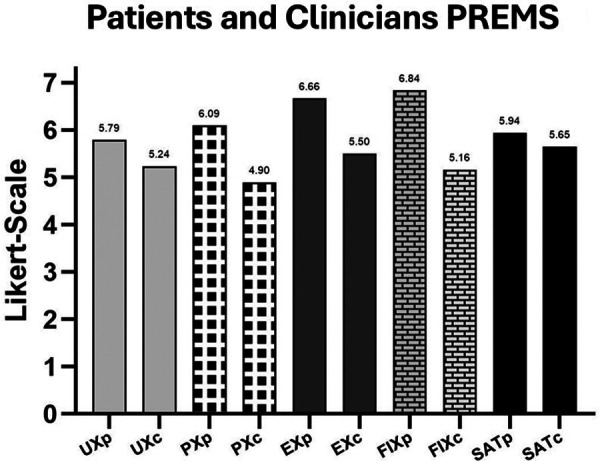
Mean PREMs cluster scores (1–7 Likert scale) by participant type (patients and clinicians; subscripts **p** and **c**).

When disaggregated by participant type (Figure 6), clinicians generally scored slightly lower than patients, with the largest difference in FIX and the closest alignment in SAT. Further insights are reported in Section 3.2.

### 3.2 Qualitative feedback analysis

Qualitative responses to item 27 were analysed using inductive thematic analysis following Braun and Clarke [32]. All free-text responses were imported into NVivo 15 for systematic coding and data management. The lead researcher conducted the initial coding, which was reviewed and refined with two additional researchers to support interpretive consistency. Codes were iteratively grouped into thematic categories corresponding to the five PREMs clusters (UX, PX, EX, FIX and SAT). This process enabled the identification of meaningful patterns across participants’ comments without applying a predefined framework. Similar qualitative approaches have been used in digital health feasibility research, such as the study by Jiménez-Díaz et al. [33].

A total of sixteen participants (eleven patients and five clinicians) submitted comments. All comments were originally collected in Spanish or Catalan and translated into English by the research team for reporting in this paper.

**Figure 7:**
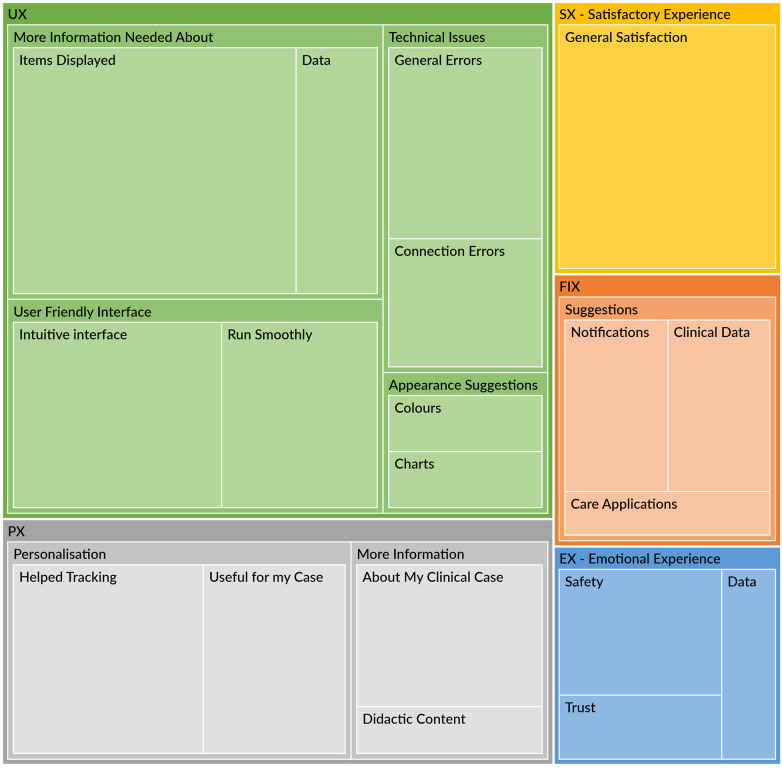
Hierarchical map of themes from open-ended PREMs responses, organised into five domains (UX, PX, EX, FIX, and SAT) and their subthemes. Box size reflects the relative frequency of each theme.

Thematic distribution of participant feedback is presented in the Qualitative Hierarchy Chart (Figure 7), where box size reflects the relative frequency of themes. Overall, UX was the most frequently discussed domain, with comments mainly referring to clarity of on-screen information, minor technical issues, and usability. PX and EX comments more often addressed perceived support for self-management and feelings of comfort and safety.

**Figure 8:**
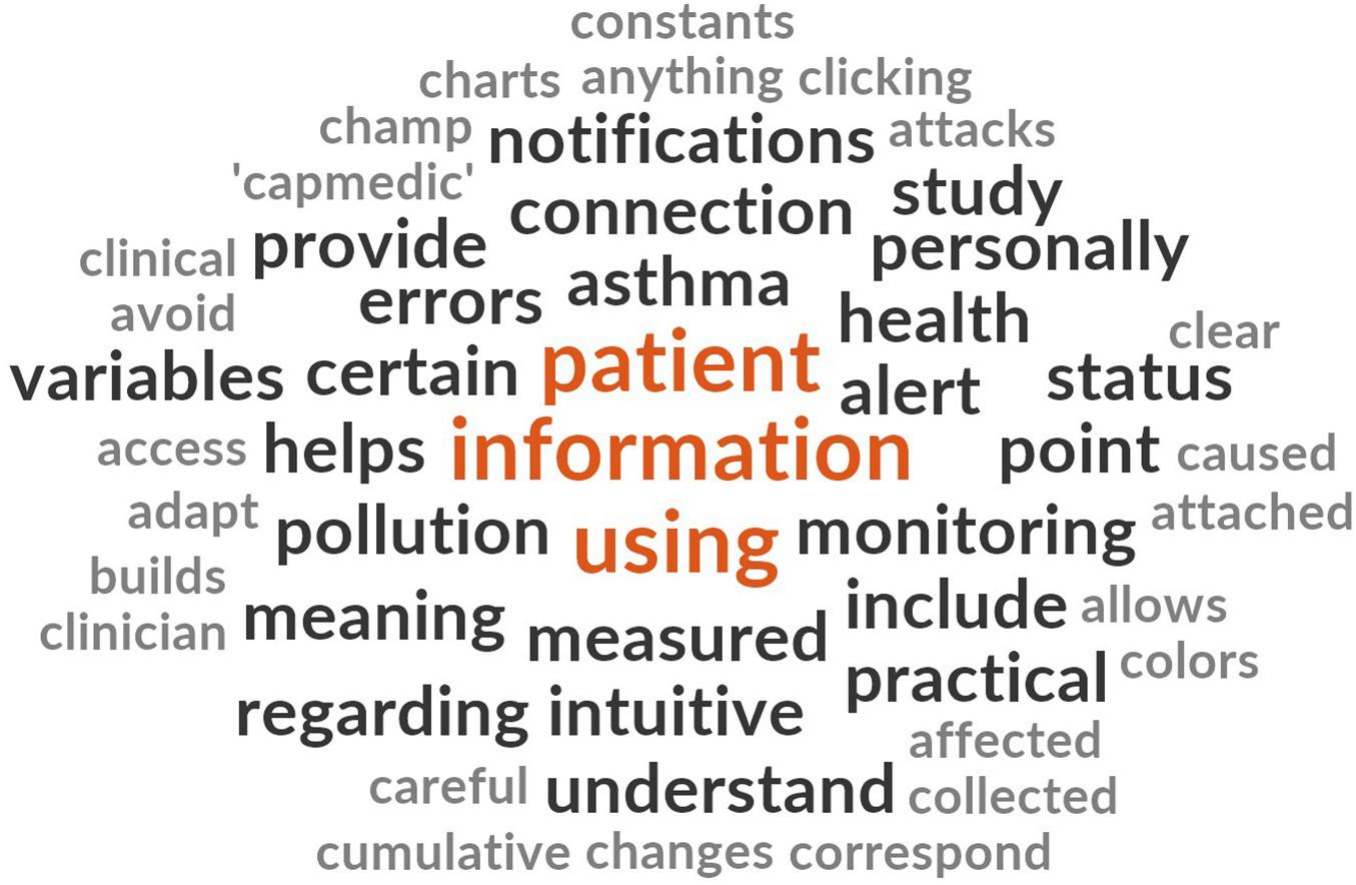
Word cloud from all open-ended PREMs responses. Word size reflects term frequency, highlighting recurrent concepts related to information needs, monitoring, and pollution exposure.

To further explore the language used in responses, a word frequency cloud was generated using all comments from both patients and clinicians (Figure 8). Word size reflects term frequency and provides a rapid overview of salient terms, complementing the thematic map in Figure 7 [34]. The most commonly used words included “information,” “patients,” and “using,” reinforcing the demand for improved clarity and more comprehensive content, particularly within the UX domain.

**Figure 9:**
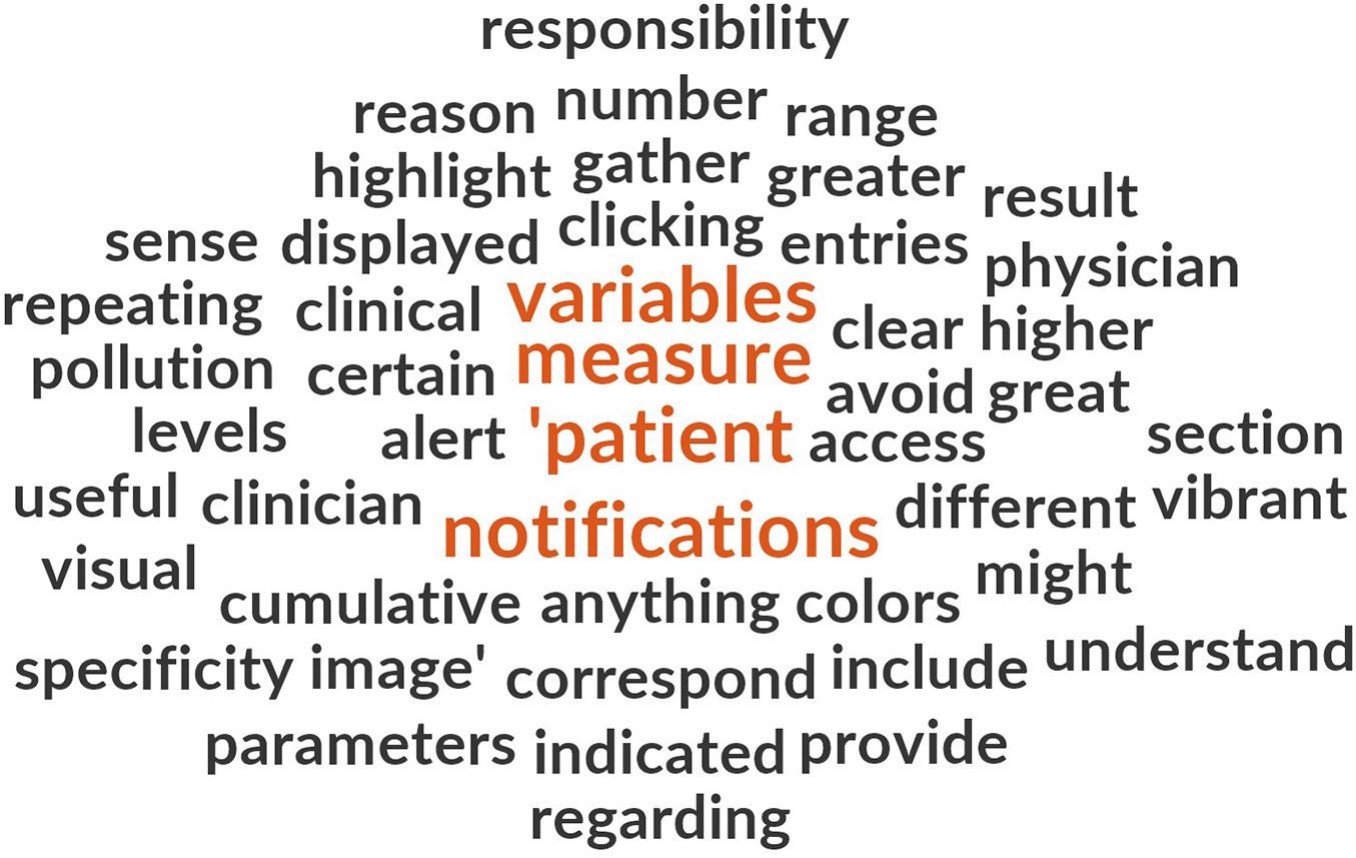
Word cloud from clinicians’ open-ended PREMs responses. Word size reflects term frequency, highlighting a focus on measurement, data interpretation, and notifications.

**Figure 10:**
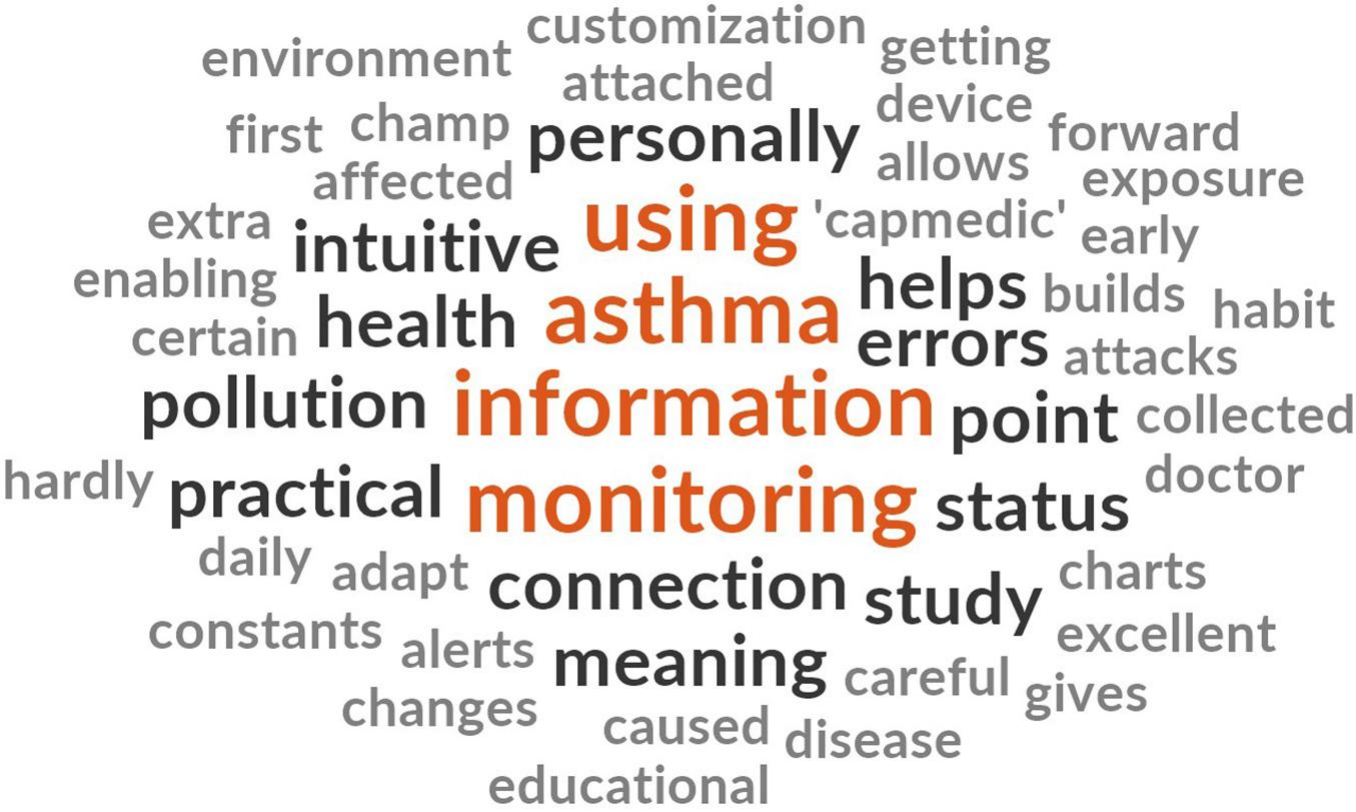
Word cloud from patients’ open-ended PREMs responses. Word size reflects term frequency, highlighting themes related to information needs, asthma management, monitoring, and pollution exposure.

Disaggregated analysis of patient and clinician comments (Figures 9, 10, respectively) revealed notable differences in thematic focus. Clinicians more frequently referred to variables such as “measures,” “patients,” “notifications,” and “data,” emphasizing system integration and data monitoring capacity. In contrast, patients more often used terms such as “asthma,” “information,” “monitoring,” and “using,” indicating a stronger focus on personal disease management and user interaction.

Overall, qualitative feedback complemented the quantitative PREMs results by clarifying user priorities and areas for refinement, particularly regarding guidance, personalisation, and minor technical issues.

## 4 Discussion

The findings from this EFS indicate an overall positive reception of the eHME among both patients and clinicians, reflected in consistently high PREMs scores across the five domains (UX, PX, EX, FIX, and SAT). Together, these results support the feasibility and acceptability of integrating physiological, environmental, and user-reported experiential data within a single digital ecosystem for moderate and severe asthma.

The lower average score for the UX cluster suggests that certain aspects of the user experience require improvement. Qualitative feedback helped explain this pattern, with participants frequently pointing to the need for clearer explanations of on-screen information and reporting occasional technical issues, including Wi-Fi or Bluetooth connectivity problems and difficulties among users with limited digital skills. These concerns align with the lower ratings for items Q13 and Q18, related to navigation and the need for written instructions. Addressing these needs through stronger onboarding and in-app guidance, such as short tutorials or clearer navigation cues, could increase user confidence and reduce confusion, particularly for less digitally experienced participants.

Analysis of the FIX cluster revealed the greatest divergence between patients and clinicians. Patients expressed strong interest in future implementation, whereas clinicians were more cautious, a pattern also reflected in the qualitative findings and consistent with literature on resistance to innovation in clinical settings [35]. Promoting adoption may therefore require closer involvement of professionals in iterative refinement, alongside co-production approaches in which patients contribute actively to implementation strategies [36]. From a development perspective, this gap highlights the need to strengthen clinically oriented functionalities (e.g., clearer data integration and workflow fit) while preserving the patient-centred elements that supported comfort, clarity, and ease of use.

As with any EFS, several limitations must be acknowledged. The small sample size, short follow-up period, and possible self-selection bias toward digitally literate individuals limit the applicability of the findings to other settings. Nevertheless, feedback from both user groups identified clear areas for improvement, particularly regarding information accessibility, onboarding support, and notification customisation, which will require further platform personalisation. The data streams generated by the eHME may enable future data-driven enhancements, including artificial intelligence and machine learning approaches to support personalised recommendations or alerts. However, this EFS dataset is not suitable for robust model development or validation, which would require larger samples. Finally, although the questionnaire was translated into Spanish and Catalan and adapted to the clinical context, the translations were not formally validated, and this should be considered when interpreting the results.

The combination of structured PREMs and inductive qualitative feedback allowed for a richer understanding of how users engaged with the platform in real-life conditions. This is consistent with recent digital health feasibility research that uses qualitative methods to explore user experience and inform iterative refinement of interventions, such as the study by Jiménez Díaz et al., who conducted in-depth interviews to evaluate a mobile health programme for caregivers and identify opportunities for improvement [33]. In particular, qualitative findings highlighted specific aspects of user trust, expectations, and perceived utility that may not be captured through Likert scale items alone. Combining structured PREMs with inductive qualitative analysis can therefore offer a more holistic view of implementation feasibility and user-centred innovation.

Looking ahead, the strong intent expressed by participants to adopt the eHME in routine clinical practice supports further scale-up and integration into standard asthma care, and opens opportunities to extend its adaptation to other areas such as mental health and wellbeing support. Continued refinement based on user feedback, including enhanced personalisation, interoperability with health systems, and expanded language support, will be essential.

In summary, this study provides strong preliminary evidence supporting the feasibility and acceptability of a patient-centred, technology-enabled approach to moderate and severe asthma care. Although further validation through larger, controlled studies is necessary, the integration of clinical, environmental, and user-reported experiential data from both patients and clinicians shows significant potential as a model for future digital health interventions. These findings offer a solid starting point for future research focused on long-term outcomes, cost-effectiveness, and the broader application of this approach to asthma management and other chronic disease contexts, including potential extensions to mental health care.

## Data Availability

The raw data supporting the conclusions of this article will be made available by the authors on request.
